# The morph as a minimal linguistic form

**DOI:** 10.1007/s11525-020-09355-5

**Published:** 2020-05-06

**Authors:** Martin Haspelmath

**Affiliations:** 1 MPI for the Science of Human History, Jena, Germany; 2 Leipzig University, Leipzig, Germany

**Keywords:** Terminology, Morph, Morpheme, Vocabulary item

## Abstract

This paper makes a terminological proposal: that the old term *morph* can be used for a minimal linguistic form. Many linguists (not only morphologists) need such a term, because we often refer to minimal linguistic forms, but the various terms used by linguists in roughly this meaning are either unclear or do not refer to forms. The term “morpheme” has three rather different meanings, and other terms such as “vocabulary item” are too abstract. The term “morph” can be used as the basis for defining other widely used terms such as “root”, “prefix”, and “suffix”, which are currently often defined as kinds of “morphemes”. It can also serve as the basis for a clearer definition of suppletion (involving suppletive morph sets) and morph variants, thus avoiding the confusions surrounding the term “allomorph(y)”.

## The morph: a basic term of general morphosyntax

In this short paper, I make a terminological proposal for general linguistics: The term *morph* should be defined as in (1), as a minimal linguistic form (i.e. a minimal pairing of syntacticosemantic content and a string of phonological segments), because this is a very useful concept that needs a term, and there is no other term that would be suitable. It is the basis for the definitions of *affix*, *prefix*, *suffix*, *root* and other frequently used terms, and is thus a very important basic term.

1. A morph is a minimal linguistic form.

All linguists are familiar with the term *morpheme*, and this term is sometimes (or even often) used in the sense of a minimal form, but it has two other prominent senses that are fairly different, so it is not suitable as an unambiguous term for a minimal form (see §3).

I emphasize that this paper is meant as a methodological contribution, and makes no larger claims, either about how particular morphosyntactic patterns should be described or how they could be explained. I merely make the presupposition that a few basic technical terms should be used consistently, just as a set of basic sounds are rendered consistently by IPA symbols throughout the discipline. Implicitly, this presupposition is widely shared, because many authors use technical terms without defining them, and this would not work satisfactorily for scientific purposes unless some basic terms were understood in the same way by everyone. (Consistent terminology is sometimes thought to be more difficult to achieve in morphosyntax than consistent use of symbols in segmental phonology, but there is no intrinsic reason for this; see Haspelmath 2020.)

Of course, many phenomena in particular languages are special and need special terms (such as “weak declension” in German, or “soft mutation” in Welsh, or “conjunct order” in Algonquian). Here I am dealing with terms and concepts of general linguistics that could in principle be applied in any language, and that are indispensable for comparative purposes. The definitions of general terms therefore cannot make any reference to language-particular phenomena.

## Examples of morphs

Before discussing other terms and the research tradition, let us consider a few concrete examples of morphs, and examples of the use of the basic term *morph* in definitions of other important terms. Examples of morphs are given in (2)–(4) (with glosses in brackets), where some of the morphs are linked to adjacent morphs by hyphens (reflecting the conventional spelling of several adjacent morphs as a single word).

(2) English
  **Table:** 

*cat*-*s*	[cat-pl]
*play*-*ed*	[play-pst]

(3) Japanese
  **Table:** 

*neko*	[cat]
*kan*-*da*	[bite-pst]

(4) Yauyos Quechua (Shimelman 2017: 34-35)
  **Table:** 

*allqu*-*kuna*	[dog-pl]
*yatra*-*rqa*	[live-pst]

Examples of technical terms that are defined in terms of ‘morph’ are *root* and *affix*. Two possible definitions are given in (5) and (6). In the examples in (2)–(4), the roots are underlined, and the affixes are not underlined.^1^

(5) A root is a morph that denotes a thing, an action, or a property.

(6) An affix is a bound morph that is not a root and that cannot occur on roots of different root classes.

My proposed definitions of *root* and *affix* may raise objections, but I will not defend them here (see Haspelmath 2012 for the definition of *root*). I assume that the least controversial aspect of the definitions is that affixes and roots are kinds of minimal forms, i.e. kinds of morphs.^2^ This is the point that is important for the current paper: We need the term *morph* in order to have definitions of other terms that we use all the time and that should have a consistent meaning across the discipline.

In the literature, roots and affixes are instead often treated as kinds of “morphemes”, as illustrated by the quotations in (7).

(7)
  - an affix is a “bound morpheme that cannot function as a word on its own” (Booij 2005: 9)
  - “Bound morphemes are usually classified into roots and affixes. Roots convey lexical meaning and affixes provide additional specification.” (Aikhenvald 2007: 38)
  - “an affix is a morpheme that is attached to a word stem to form a new word or word form” (Wikipedia in 2020, under “affix”)^3^

It seems that in these definitions, when the authors say “morpheme”, they mean a minimal form, i.e. what I suggest should be called *morph*. But the term “morpheme” has at least three different meanings in the current literature, so it is hardly suitable for the sense of a minimal form that is required to define roots and affixes. I will discuss these meanings in the next section.

It may be noted that the proposal to use the term *morph* in this sense is not quite novel, and indeed, the term is over 70 years old (see n. 6). But it has rarely been used in the intended sense in the last half century, so it makes good sense to remind linguists of a useful term that can serve to give clear and simple definitions of other terms that they use all the time.

## The term “morpheme”

The term *morpheme* was coined by Jan Baudouin de Courtenay in 1880, and has become widely known through its use in Bloomfield’s *Language* (1933). However, it has not been used consistently over the years. The inconsistent usage was described in some detail by Mugdan (1986) for the first hundred years of the term’s existence,^4^ and the situation has not improved since then. Carstairs-McCarthy (2005: 22) suggests that perhaps “the term ‘morpheme’ has hindered rather than helped our understanding of how morphology works”. The term has been used in one of the three senses in (8), which are often not properly distinguished.

(8)
  - morpheme 1:
    a minimal form (= a morph)
  - morpheme 2:
    a set of minimal forms with identical syntacticosemantic content
    (= a set of homosemous minimal forms, see n. 4)
  - morpheme 3
    a minimal element of (morpho-)syntactic representation

The first sense can be found in definitions of types of morphs, like affix and root (as seen in the preceding section), but it is also widely found elsewhere in the literature. When a linguist needs to refer to a minimal form outside of morphological theorizing, they are very likely to call it *morpheme*. Thus, the first sense could be described as the non-technical (or colloquial) sense of the term.

The second sense is quite prominent in the literature dealing specifically with morphology, and often appears in discussions of “allomorphs”, e.g.

(9)
  - “Allomorphy is the phenomenon that a morpheme may have more than one shape.” (Booij 2005: 31)
  - “Variant forms of the same morpheme are called allomorphs.” (Kroeger 2005: 289)

For example, Kroeger (2005: 288-289) lists pairs of “allomorphs” such as these:

(10)
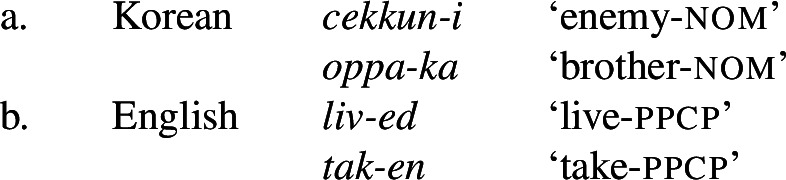

Each of these examples shows two different affixes that have the same syntacticosemantic content (the same grammatical meaning, or the same morphosyntactic features; we can also say that they are *homosemous*).^5^ Kroeger treats them as “allomorphs” of the same morpheme, and this usage is not uncommon (see §8 below). The morpheme in this sense is thus an abstract entity: it is not a form, but a set of minimal forms (in other words, a set of morphs) (this set-based definition is the one used by Hockett 1947: 322, and it is also very clear in the detailed discussion provided by Mel’čuk 2006: 384–397).

If one uses the term in this second sense, one can no longer felicitously say that a morpheme has a certain shape (e.g. “the morpheme *-i*”), or that some meaning is expressed by a morpheme, or that a word is divided into morphemes, or that several morphemes occur in a certain order, because the morpheme in this sense is not a concrete form. Of course, linguists say these things all the time, but when they do, they necessarily use “morpheme” in sense 1 (i.e. in the sense of ‘morph’). But this sense is incompatible with sense 2, and it is not coherent to say at the same time that a morpheme has a concrete shape and that it has several different “allomorphs” (like *-en*/*-ed* in English, cf. (10b)). Thus, one has to choose between one of the meanings if one wants to use consistent terminology.^6^

Since it is identical syntacticosemantic content that is shared by the forms, some authors call this content itself the *morpheme*, as in the following quotation:

“Clearly the past tense form of *loved* consists of two morphemes, the verb-stem *love* and a grammatical morpheme which we can call *Past*, and it’s not too hard to draw a line between them. But the past-tense form *took* must likewise consist of two morphemes, the verb-stem *take* and the morpheme *Past*, yet this time we can’t draw a neat line at all: the two morphemes are just wrapped up in a single bundle, and we have to appeal to a more abstract level of representation to show that *took* is really *take* plus *Past*.” (Trask 1999)

Such “abstract morphemes” (“minimal elements of (morpho-)syntactic representation”, sense 3 above) have recently become prominent in the Distributed Morphology (DM) tradition, following Halle and Marantz (1993), and Embick’s (2015) general book about DM is titled *The morpheme*. As Mugdan (1986: 36) notes, the notion of abstract morphemes actually goes back to the 1960s (e.g. Bierwisch 1967; Chomsky 1965). In the 1960s and 1970s, they were often alternatively called “formatives”, but this term seems to have largely gone out of use.

To summarize, traditions that use the term “morpheme” can treat the two English plural forms *book-s* and *ox-en* in three different ways: They can say (i) that -*s* and *-en* are two different (but homosemous) morphemes expressing plural meaning (sense 1), (ii) that -*s* and *-en* are members of the same English morpheme (sense 2; designated {Plural}, or {-*s*, -*en*}, using curly brackets for sets), or (iii) that there is an abstract morpheme [plural] that may be realized by different exponents (-*s* and *-en*) (sense 3).

Because of this multiplicity of meanings of the term “morpheme”, I do not think that the term can be salvaged for future use in technical contexts. It will no doubt continue to be used colloquially (mostly in sense 1), but it seems best to avoid it in technical usage if one wants to be understood more widely. In its colloquial sense, it can be easily replaced by *morph* by authors who value precision and a broad readership.

We began in §2 with definitions of the terms *affix* (which is the basis for *suffix* and *prefix*) and *root*. Having simple definitions of these is important, because the terms *suffix* and *prefix* are very widely used, and in fact my original motivation for writing this paper was that I was very dissatisfied with many of the definitions of these commonly used terms in the literature.

Could one perhaps define an affix as a kind of “morpheme” in sense 2 or 3 in (8)? It seems clear that the answer is no, because we would not want to say that there is a “Korean suffix -*i*/*-ka*”, implying that -*i* is the same suffix as *-ka*, or that English has “a suffix *-en*/*-ed*” (whereas we could say that *-i* and *-ka*belong to the same morpheme {Nominative}, in sense 2, or that they realize the same abstract morpheme [nom], in sense 3).^7^ Thus, affixes and roots are generally understood as special kinds of morphs, not as “morphemes” (unless one uses this term in sense 1, to refer to morphs).

## More on forms (or expressions, or signs)

The definition of *morph* that was given above contains the term *form* (“a morph is a minimal linguistic form”), which is not remarkable (because linguists talk about forms all the time), but which nevertheless deserves some discussion.

A form is a recurrent pairing of content and segmental shape: “Every language consists of a number of signals, *linguistic forms*. Each linguistic form is a fixed combination of signaling-units, the *phonemes* …We assume that each linguistic form has a constant and definite meaning” (Bloomfield 1933: 158). The elements in (11) are forms of the English language.

(11)
  - *The cats are not on the mat*.
  - *I’m hungry*. (Bloomfield 1933: 158)
  - *the cat*
  - *cats*
  *-s*

Sentence fragments such as *not on the* are not forms because they do not have a coherent meaning, but all sentences, clauses, phrases, words and morphs are also forms.

This is how linguists talk all the time, although they rarely reflect on the fundamental term *form*. An alternative synonymous term is *expression*.^8^ One might also use the term *sign* for a pairing of content and segmental shape, although few linguists do so on an everyday basis (one might talk about “nominals, person prefixes and other object forms/expressions”, but one would not say “object signs” in this context).^9^

In addition to segmental shape, suprasegmental shape (pitch, intensity, length) is of course important in all languages as well. And syntacticosemantic content may also be expressed by vowel apophony or consonant mutation, by apotony, or by reduplication, as illustrated by the pairs in (12).

(12)
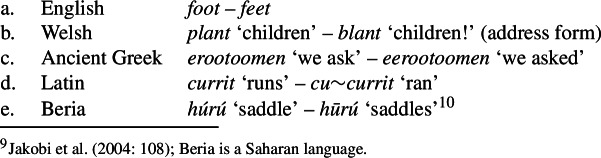

Such nonconcatenative pairs of forms have often been highlighted in order to demonstrate that a simple concatenative view of morphology is insufficient (e.g. Hockett 1947; Anderson 2015: §1.4). However, such cases do not have any consequence for the definition of the terms *a form* and *a morph*. Vowel and consonant changes, suprasegmental changes and reduplication are normally thought of as processes, not as forms, and hence not as morphs. Moreover, morphologists sometimes say that a “morpheme” may be realized by zero, but again, it should be clear that a form cannot be zero (because it is a pairing of a shape and a meaning). Thus, morphs (as forms) must be segmental.^11^

Morphs must also be continuous, because otherwise there would be no limits on the kinds of “discontinuous morphs” that one might posit (for example, what stops one from saying that *have* ...*-en* is a single discontinuous Perfect morph in English?). Thus, a “circumfix” is really a construction containing both a prefix and a suffix (rather than a single affix). And similarly, the non-infix part of an infixed form cannot be a morph.^12^

The term *morphology* may suggest that the term *morph* should be used in a wider sense, including more than segmental forms. However, nobody has suggested that morphology should be defined as the study of morphs. Morphology was originally defined as the study of linguistic forms, but as syntax became more prominent, morphology became restricted to “word forms” (though these are difficult to delimit from phrasal forms; see §6). It seems that nobody would deny that concatenating morphs is what many morphological and syntactic rules do (or can be thought of as doing), so even if the term *morphology* is not quite transparent, is it not completely inappropriate either.

In addition to nonconcatenative aspects of shape, linguists have also sometimes identified meaningless strings, sometimes called “empty morphs” (e.g. -*th*- in English *far-th-er*). Such strings are not morphs by the current definition unless they are said to have some syntacticosemantic content (e.g. providing a suitable stem for an affix to attach to). But note that the definition given here does not require that all elements of an utterance belong to some morph (just as not every morph is either an affix or a root, cf. note 1). Analyses must eventually be complete, but technical terms need not cover everything.

Thus, morphs are segmental, as they are kinds of forms, which are defined as having a (continuous) segmental shape. Nonsegmental aspects of shape do of course also have an important role in morphosyntax, but they will have to be captured by other terms (not discussed in this short paper).^13^ So while the definition in (1) is strongly inspired by the Bloomfieldian tradition (where careful general terminology was considered important), the present context is rather different: The goal is not to achieve complete and elegant description (i.e. there is no “total accountability”, as in Hockett 1947: 332), but clarity and consistency of some basic concepts and terms.

## Minimality

A morph is defined as a minimal form, which means that it does not consist of other morphs.

Now of course, whether a form is minimal or can be further divided into smaller forms with their own content is not always clear. Linguists often disagree about morphological segmentation, e.g. whether English *him* and *them* can be analyzed as containing an accusative suffix (*hi-m, the-m*) or should be treated as unanalyzable forms, like *her* and *us*. Such questions will never go away, and they will always be answered differently by different linguists (and presumably also differently by different speakers of a language).^14^ But the existence of such borderline cases does not affect the definition of the term *morph*.^15^

What is important to realize is that minimality is not the same as “simplicity”. Linguistic forms are sometimes said to be “complex” even when they cannot be segmented into two or more morphs. Famously, Bloomfield (1933: 161) defined the *morpheme* as a “simple form”:

“A linguistic form which bears no partial phonetic-semantic resemblance to any other form, is a *simple* form or *morpheme*.”

But morphs such as English *feet* or Welsh *blant* (cf. (12a-b) above) are not “simple” in this sense, because they are semantically and formally similar to other forms. In English, there are at least a few other pairs like *goose* – *geese* and *woman* – *women*, and in Welsh the pattern is even productive. Thus, these forms are generally regarded as “morphologically complex”, but they cannot be segmented,^16^ so they are distinct minimal forms (i.e. distinct morphs).

Of course, the contributions made by the suprasegmental and segmental changes seen in (12) are in an important way parallel to the contributions made by grammatical markers (= grammatical morphs), and one eventually needs to have a way of talking about this parallel (e.g. in terms of morphological constructions, or realization rules). But recall from §1 that I am not trying to make a contribution to methods of elegant description, let alone theories of mental morphosyntactic knowledge. This paper is about clear terminology, and in this context, a notion of a “process morph(eme)” would not be compatible with terminological clarity. A “process” is not a form, and since a morph is a form, it cannot be a process.

For theories that aim for elegant description, it may well make sense to postulate additional phenomena such as “circumfixes” (see §4), or even “synaffixes” (like English *-abil-ity*, Bauer 1988) or “morphologically complex affixes” (cf. Stump’s (2017) “micromorphology hypothesis”). But it would be better to choose other terms for these phenomena, because these elements cannot be affixes (i.e. types of morphs, (6) above) in any standard sense.

Some theories that aim for elegant description and/or description of speakers’ mental representations have claimed that linguists should not try to isolate minimal forms at all. For example, Anderson (1992, 2015) prefers to look at all of morphology in terms of rules rather than minimal forms, and Bochner (1993), Ford et al. (1997), Stump (2001), and Blevins (2016) have proposed similar theories. Most recently, Jackendoff and Audring (2020) have proposed that all of morphology can be captured by interlinked schemas and sister schemas of different generality. These theories are very attractive, especially if they also extend to all other morphosyntactic patterns (like Jackendoff’s theory does). However, they are not very relevant for the everyday practice of linguists, who cannot use complex Paradigm Function or Relational Morphology notation when they merely want to discuss, say, the conditions on the use of accusative case-markers in a range of Iranic languages. De facto, all linguists work with forms/expressions that may be smaller than an utterance (sentences, clauses, phrases, and so on), and definitions of many such terms for larger form types must ultimately be based on minimal forms.^17^

## Morphs and morphosyntax

Terms like *affix* and *morpheme* are most often discussed by linguists specializing in morphology, while others tend to simply presuppose that they have a clear meaning. In the present context, it is important that the term *morph* is a fundamental concept for all of morphosyntax, so that it cannot be discussed only against the background of the favorite topics of morphologists.

As has been pointed out repeatedly (e.g. Haspelmath 2011; Bruening 2018; Bauer 2019: 2), there is no clear way of delimiting morphology from syntax. Clitics cannot be clearly delimited from inflectional affixes (e.g. Spencer and Luís 2012: §9.3), and compounds cannot be clearly delimited from phrases (e.g. Lieber and Štekauer 2009). Moreover, inflection cannot be clearly delimited from derivation (e.g. Plank 1994).

An important feature of the definition of *morph* as proposed in this paper is that it is not affected by these difficulties of delimitation. It merely relies on the basic concepts of a ‘segmental form’ (§4) and of ‘minimality’ (§5), which are necessary anyway. They do not rely on a syntax-morphology distinction or an inflection-derivation distinction, which may turn out to be not more than remnants from an older descriptive tradition.

This advantage can be seen when comparing it with another definition, by Crysmann and Bonami (2016: 314)

“We use MORPH as a cover term for recurrent partials that are identifiable within the paradigm of one lexeme. On this definition there are only three kinds of morphs: stems, affixal exponents, and discontinuous stem formatives. The fact that stems of derived lexemes can be further segmented will be ignored, as this plays no role in the current discussion.”

This definition presupposes the terms *lexeme* and *paradigm* (which seem to rely on a notion of inflection), as well as perhaps the notion “stem” (which is not generally clear, unless it means ‘whatever remains after subtracting all inflectional affixes’, where it again presupposes a notion of inflection). I do not mean this observation as a criticism of Crysmann and Bonami’s specific theory (where these notions may well have a very clear meaning), but as an illustration of my claim that the field of linguistics as a whole will find a simple and presupposition-free definition of the term *morph* very useful.

## Morph variants

In addition to being clear about forms and minimality, we need to say more about phonological variability. It is well-known that morphs often have phonological variants, as is illustrated in (13).

(13)
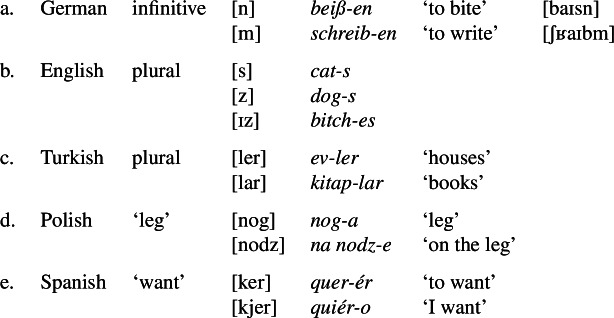

In these cases, it is perfectly normal to say, for example, that [s] and [z] in English are the same suffix, or that [ker] and [kjer] in Spanish are the same root. Thus, linguists do not think that an affix or a root (and hence a morph) must always have exactly the same shape. However, the shape variation must be of a phonological nature. If two different segment sequences are not phonologically similar at all, they will be regarded as different morphs even if they are homosemous (semantically equivalent), as we saw in (10a-b). This applies both to affixes and to roots (see §8 below on suppletive affixes and roots).

As in the case of minimality (§5), linguists will often take different decisions concerning the distinction between phonological variants of the same morph and different morphs. When an alternation is very regular and occurs in a variety of different environments, it will normally be treated as phonological, even if it is not very natural phonetically (this applies to the Polish alternation between [g] and [dz], for example). When an alternation applies only to few cases but is phonetically natural, it is also usually treated as phonological (as in the case of English [s]/[z]/[ɪz]). When it is neither phonetically natural nor regular (e.g. English *is* vs. *are*), then everyone treats the two segment sequences as different morphs, even if the forms are descended from the same morph at an earlier stage. Thus, there is very broad agreement on the principle of distinguishing between morph variants and different homosemous morphs (see §8 below), even if in many individual cases, judgments may differ.

In §3 above, I said that English *-en* and *-ed* (in (10b)) would not be said to be the same suffix. By contrast, I think that we would say that *-s* and *-es* (in *dogs* and *bitches*) are the same suffix, or variants of the same suffix, just as we would say that English *analyze* is the same word as *analyse*, and that both are spelling variants of the same verb. This identity relationship might be underlined by deriving both *-s* and *-es* from the same abstract underlying form, but this is not necessary in order to recognize their identity (after all, there is no abstract underlying form for the spelling variants *analyse*/*analyze* either).

## Suppletive morph sets

A morph is sometimes homosemous (see note 5) with another morph and occurs in a complementary morphosyntactic distribution. For example, Latin has the root *fer-* ‘carry’ in the Present tense, but *tul-* ‘carry’ in the Perfect:

(14) Latin
  - *fer-o* ‘I carry’, *fer-s* ‘you carry’, etc.
  - *tul-i* ‘I carried’, *tul-isti* ‘you carried’, etc.

Such sets of morphs are called suppletive morph sets. Linguists usually say that such cases involve different roots, so *fer-* and *tul-* are different morphs.^18^

Suppletive morph sets that occur in complementary distribution are also found in inflection. For example, German has a variety of different plural morphs, which are sometimes conditioned by gender or derivational suffix, but are often distributed arbitrarily, e.g. *Tag*/*Tag-e* ‘day(s)’, *Lied*/*Lied-er* ‘song(s)’, *Staat*/*Staat-en* ‘state(s)’, *Wrack*/*Wrack-s* ‘shipwreck’. In Hungarian, a second-person singular subject is usually indicated in verbs by the morph *-sz*, but by the homosemous morph *-el* when the verb stem ends in a sibilant (Carstairs 1988):^19^

(15)
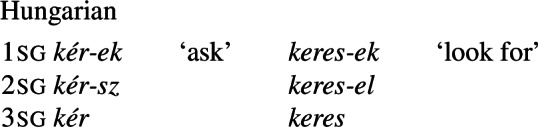

Again, in such cases linguists usually say that there are different affixes (we already saw another example of this phenomenon in (9a) above). Thus, suppletion involves different morphs, i.e. a set of different (but homosemous) forms.^20^

The term *morpheme* would not be appropriate for the different elements in sets like *fer-*/*tul-* and *-sz*/*-el*, because it has often been used in an abstract sense (sense 3 in 8c), to refer to whatever content the suppletive morphs share (e.g. Lyons 1968: 182-183: “*worse* is composed of two morphemes, one of which it shares with *bad*, and the other of which it shares with *taller*, *bigger*, *nicer* etc.).

As noted in the preceding section, the boundaries between suppletion and (morpho)phonological alternations are not sharp (e.g. Dressler 1985; Kiparsky 1996; Plank and Vincent 2019). In *bad*/*worse*, we clearly have a suppletive morph set, and in cases like , we clearly have two variants of the same morph. But is *bett-* (in *better*) the same morph as *be-* (in *best*)? Or are they two different morphs, constituting a suppletive morph set? As in the case of minimality in §5, different linguists (and perhaps different speakers) will always give different answers,^21^ but what matters here is that there is a clear conceptual distinction: Either two shapes are variants of the same form, or they are two different forms.^22^

## “Allomorphy”: supplemorphy and variomorphy

The term “allomorph(y)” is used in scare quotes in this paper, because I do not think that it can be defined in such as way that the definition is internally consistent and at the same time very largely compatible with existing usage. Like the term “morpheme” (§3), the term “allomorph” has the problem is that it is used in rather different ways in the literature (though here I will highlight two rather than three different senses).

It appears that in the morphology textbooks, “allomorph” tends to be used in the sense of ‘morph variant’ (§6). But in the research-oriented literature, it tends to be used in the rather different sense of ‘member of a suppletive morph set’ (and the term “allomorphy” is then used in the sense of “suppletion”).

For example, Lieber (2009: 158) says in her textbook that “allomorphs are phonologically distinct variants of the same morpheme”, and Booij (2005: 31) defines “allomorphy” as “the phenomenon that a morpheme may have more than one …particular phonological form” (likewise Harley 2006: 131).^23^ The main issue that these textbooks mention is that some linguists limit the term “allomorph” to unpredictable morph variants. Thus, Booij (2005: 32-33) says that one might want to limit the term to cases like (13d-e) (e.g. Spanish *quer-*/*quier-*), where the alternation is morphophonological (not phonologically automatic), presumably because the morphological notion of “allomorph” is not really needed when there is clearly a unique underlying form from which the phonological realizations can be derived by phonological rules.^24^

In the research-oriented literature, by contrast, the term “allomorph” is typically used for a member of a suppletive morph set. Kiparsky (1996) contrasts “allomorphy” with “morpho-phonology” (cf. also Carstairs 1987), and in the same vein, Bonet and Harbour’s (2012) survey paper discusses suppletive morph sets in contrast to (morpho)phonological morph variants, noting that “others use the term ‘suppletion’ to refer to multiple underlying forms” (2012: 199).^25^

Thus, two rather different meanings are associated with the same term “allomorph”: ‘morph variant’ and ‘suppletive morph’. In view of this situation, it seems best to avoid the term (just as it is best to avoid the term “morpheme”) and to use other terms instead.^26^

Maybe one could use *supplemorphy* for a situation where there is a suppletive morph set, and *variomorphy* for a situation where a morph has several variant shapes.^27^ But while one could talk about several homosemous *supplemorphs* (e.g. English *go* and *wen-*, two homosemous morphs meaning ‘go’), the term *variomorph makes no sense (the morph variants -[d], [t] and [ɪd] of the English plural morph -*s* are not different morphs).

## Homonyms and idioms

Above I defined a morph as a minimal pairing of meaning and form, or content and shape. I already discussed two ways in which such minimal forms are not straightforward to delimit from other forms: Their minimality may be in question (e.g. in the case of English *them*, possibly segmentable into *the-m*), and their identity may be in question (e.g. in the case of *be-* in *best*, which may be identical to *bett-* in *better* or not).

In addition, of course, one may wonder whether a pair like *bank* and *bank* is a single polysemous morph or whether we are dealing with two different morphs that just happen to have the same shape (i.e. two homonyms). The same issue arises with grammatical markers: Is the Russian -*a* in *volk-a* ‘wolf (accusative)’ the same morph as -*a* in *volk-a* ‘wolf (genitive)’ (so that we are dealing with systematic syncretism), or are they two different homonymous morphs? These questions have often been discussed, but like the questions about minimality and identity, they will always be answered differently. Again, fortunately, they are not relevant for the definition of ‘morph’.

Less often discussed in the present context is the question of how to deal with complex but noncompositional expressions like *white wine*, *hard disk*, *deadline*, *spill the beans* ‘divulge a secret’, *leave no stone unturned* ‘search intensively’, or German *Handtuch* (‘towel’, mentioned in note 14). Strictly speaking, these cannot be said to consist of a combination of morphs, because we can identify a form only if it has a “definite meaning” (as Bloomfield 1933: 158 put it). But in many cases, the figurative nature of such idioms is quite transparent to the speakers (who may not even notice the lack of compositionality, e.g. in the case of *white wine*). In those cases where there is no transparency at all (e.g. *kick the bucket* ‘die’, *hot dog* ‘sausage sandwich’), it is harder to justify a treatment in terms of multiple morphs, but such cases seem to be uncommon. As in the other cases, I have nothing to say on the issue of delimitation here, which arises in any event and does not seem to be resolvable in a general way.

## Related terms: *exponent, vocabulary item, listeme*

Finally, let me briefly discuss three terms that are similar in meaning to *morph*: *exponent*, *vocabulary item*, and *listeme*.

The term *exponent* seems to be fairly old, but has become better known to linguists through Matthews (1972, 1974). In the 21st century, its popularity seems to have increased, and there are now books with “exponence” in their title (Trommer (ed.) 2012; Harris 2017). A typical use of the term *exponent* is as a minimal form that realizes a set of morphological features. However, in inflectional paradigms, there are often some cells that lack forms expressing the relevant inflectional meaning (e.g. the nominative singular in Russian nouns like *volk-Ø* ‘wolf’, contrasting with genitive singular *volk-a*, nominative plural *volk-i*, etc.). For such cases, many authors work with a notion of a “zero exponent” (e.g. Plank 1999: 282; Calabrese 2011; among many others). Moreover, in the discussion of “multiple exponence”, both a plural suffix and plural vowel change are said to be exponents (e.g. German *Hals* ‘neck’, *Häls-e* ‘necks’, Harris 2017: 1). According to the definition proposed here, a morph (or any other form) cannot be nonsegmental, so a vowel change is an “exponent” which is not a morph.

The term *vocabulary item* has also become widely used, in the Distributed-Morphology literature, and it seems to have basically the same meaning as *exponent*. While Harley and Noyer (2003: 468) say that “Vocabulary Items provide the set of phonological signals available in a language for the expression of abstract morphemes”, they include in their examples a vocabulary item that has no phonological features, and is thus not a form (or a morph) in the sense of the present proposal.

Finally, the term *listeme* (originally proposed by Di Sciullo and Williams 1987) is used extensively in Harley’s (2006) textbook in a sense similar to ‘morph’. Harley takes pains to explain that English plural -*s*/*-es* represent the same listeme, while plural -*i* (as in *alumn-us*/*alumn-i*) is a different listeme. But if a listeme is ‘anything that we need to memorize’ (and that thus needs to be listed in some mental repository), then this term is much broader than ‘morph’. Thus, idiomatic compounds like *newspaper* and *wallpaper* need to be memorized (Harley 2006: 100), and are thus listemes, but they are at the same time very transparent, so one would not say that they are morphs (as discussed in §10). As Jackendoff (1997: Chaps. 5–6) discusses extensively in the context of multi-word expressions, the relationship between what is listed mentally and what is described by rules is not straightforward, even if we assume (counterfactually) that our linguistic knowledge is stored nonredundantly. Thus, a notion of listeme is certainly different from *morph* as defined here.^28^

## Concluding remarks

This paper has discussed a range of different terms that are used widely in morphosyntax, using a text genre that is almost unknown in contemporary linguistics (but see Mel’čuk 1982, 2006; Mugdan 1986, 2015 for similar work on technical terms). But in view of the fact that the terminology that we use is often inconsistent and unclear, I hope that my readers find this exercise useful.

Many morphologists prefer to direct their energies into rather different directions, e.g. attempting to describe particular phenomena in a highly elegant way (and hoping that the approach will eventually generalize), or proposing ambitious architectures intended to reflect the mental organization of linguistic knowledge in all speakers and all languages. If we made good progress in these (“theoretical”) efforts, we might dismiss terminological discussion as a minor and irrelevant aspect of methodology, because true discoveries should be apparent even without clear terms. But if progress in the field is not expected to be fast, then we might as well devote some energy to consistent terminology, so that at least the definitions of basic terms in our textbooks (and thus in derivative works like Wikipedia) will be non-circular and clear. Without clear terminology, we will keep talking past each other, and if we make real progress, we will not be able to communicate this clearly.

Finally, I should remind the readers that I am not making claims about how languages are best described. I defined ‘morph’ in terms of a segmental string, because everyone agrees that such entities may be useful for comparing languages, not because I would suggest that morphs are sufficient to describe languages (they are obviously not sufficient). Neither do I want to suggest that all minimal strings that are usefully segmented are morphs (see the brief discussion of English *-ceive* in note 15). While general theories must ultimately be complete, this methodological paper makes no attempt to provide a complete set of concepts (see also the brief discussion in note 1 of roots, affixes, and other elements for which there exists no general term). It merely provides a simple and clear definition of the term *morph* (as a minimal linguistic form), for the purposes of general linguistics, which will probably be useful for many linguists.

## Footnotes

1 The morphs in (2)–(4) are all roots or affixes, but languages may also have morphs that are neither roots nor affixes, often called “particles” or “clitics”. Some elements called “determiners”, “adpositions” or “auxiliaries” also fall in this group. There is no widely used general term for non-affix non-root morphs.

2 Readers who are skeptical of the definitions in (5) and (6) are invited to propose better ones. After examining a large number of attempted definitions over two decades, my preliminary conclusion is that these are the best available definitions (for more discussion of the term *affix*, see Haspelmath 2021).

3 https://en.wikipedia.org/wiki/Affix, accessed on 2020-04-14.

4 Matthews (1991: 120) comes to a similar conclusion (apparently independently of Mugdan’s paper, which he does not cite): “The term ‘morpheme’ has a complex history and it is not always clear in which sense it is being used…Present usage is confused.“

5 The term *homosemy* was proposed by Harley (2006: 146) to refer to the full identity of meaning or syntacticosemantic content (in contrast to *synonymy*, which is typically used for semantically similar word pairs such as *big*/*large*, or *stop*/*cease*, which are not in complementary distribution).

6 The problem of inconsistency was also present in the early usage of the term “morpheme“ by Baudouin de Courtenay and by Bloomfield (as noted by Mugdan 1986). It was solved in the Bloomfieldian tradition through the introduction of the distinction between “morpheme“ (for the set of forms, meaning 2) and “morph“ (for the minimal form, meaning 1) by Hockett (1947) (an earlier proposal was Harris’s (1942) “morpheme alternant“). For some reason, this distinction was not taken up by most of the literature since the 1970s, although it is still found in Matthews (1974, 1991) and Mel’čuk (2006). It also occasionally occurs in more recent works (e.g. Spencer 2013: 238, Blevins 2016: 25, Crysmann and Bonami 2016, Harris 2017: 20), though rarely systematically.

7 Likewise, using the term “allomorph“ (see §8), we would not say that Korean *-i* and *-ka* are “allomorphs of the same suffix“.

8 To be sure, term *expression* is much more often used for longer forms/expressions (perhaps comprising several words), while the term *form* tends to be used in particular for word-forms (e.g. (11d)). But I am not aware of any definitions of these terms that restrict them in these ways.

9 Note that the basic term *form* is homonymous with another basic term: *form* in contrasts such as “meaning/content vs. form”. If the context does not make it clear, it is therefore better to oppose meaning/content and *shape*, as is done in the text.

11 Another term that is sometimes used in this connection is “unit”, as when a morph(eme) is said to be “the smallest meaningful unit”. However, this is an extremely vague term (like “element”), and it is unclear what falls under it (perhaps all kinds of processes and zeroes).

12 Likewise, introflective patterns of the Semitic type (sometimes called “root-and-pattern morphology”) cannot be said to be composed of morphs with intercalated vowel patterns. In the Arabic form *katab-tu* ‘I wrote’, the two *a* elements might be regarded as two infixes (jointly signaling Perfect Active), but *k-t-b* is not a segment sequence, i.e. not a morph (and hence not a root, even though the term *root* originates in Semitic linguistics).

13 Mel’čuk (2006) defines morphs as “minimal signs“ (see also Mugdan 2015: §2.2), but he uses the term “sign” for a variety of different processes (including vowel change phenomena), so that his definition ends up being highly complex, and his “morphs” are hard to delimit. (It is not clear, for example, whether word order alternations such as question-word fronting should not also be regarded as “elementary linguistic signs“, whereas it is very clear that such operations/transformations are not forms or expressions.) In general, it seems that the only practical way to achieve terminological unification is to have straightforward definitions for simple terms. If one wants to propose more complex concepts, one should choose novel or more complex terms for them.

14 Or differently at different speaker ages (e.g. German *Handtuch* ‘towel’ is typically acquired early as a minimal form, but later in life – after learning the orthography – many speakers come to segment it into *Hand* ‘hand’ and *Tuch* ‘cloth’, even though the meaning is not transparent).

15 How one segments forms also depends on what one allows as syntacticosemantic content. Segmenting Spanish verb forms into three morphs (stems, stem vowels and endings: *habl-a-mos* ‘we speak’, *com-e-mos* ‘we eat’, etc.) may be justified by saying that the stem vowels are syntactically (or morphotactically) required. But in cases like English *-ceive* and *-duce* (cf. *perceive*/*receive*/*deceive* vs. *perception*/*reception*/*deception*, *reduce*/*deduce* vs. *reduction*/*deduction*), there is no such justification, so these would not be morphs. (A description of English could or should still segment these, of course, but they cannot be regarded as morphs for comparative purposes.)

16 Here I leave aside the possibility of segmenting *foot* and *feet* into a frame element *f-V-t* and a singular and a plural infix, respectively. (Positing a nominative *p-* and a vocative *b-* in Welsh would be even more far-fetched.)

17 And if one wants, one can probably define a morph in the terms of these theories (see Jackendoff and Audring (2020: 90) for discussion of a “smallest meaningful unit” in their perspective). But in the current paper, I focus on terms that can have the same meaning across the discipline of linguistics, i.e. on basic methodology rather than specific theories.

18 E.g. Booij (2005: 34): “The Italian verb *andare* has two suppletive roots: *and-* when the root is not stressed, and *vad-* when the root is stressed in the verbal paradigm (*vádo*, *andiámo*, etc.).”

19 Cases like (15) show that it is important not to confuse *phonological variants* with “phonologically conditioned variants“. Two phonological variants of the same morph are phonologically similar, ideally in a very regular way, but phonological conditioning may apply to suppletive morph sets (such as *-sz*/*-el*) as well (see also Paster 2009).

20 One might question the suppletive nature of a morph set on the grounds that the distribution is not completely complementary (e.g. Swedish *bättre* ‘better’, which is the usual comparative counterpart of *god* ‘good’, but it exists alongside a form *godare* with a somewhat different meaning). But again, this does not affect the definition of *suppletive*, because it is due to some vagueness in the more general term *complementary distribution*, which is an independent fact.

21 Occasionally one hears the form *bestest*, which suggests that *best* is not analyzed into *be-* and *-st* (i.e. that *good*/*bett-*/*best* are a suppletive morph triple).

22 In the literature, the notion of “suppletive allomorphy” is strongly associated with inflectional markers and roots. By contrast, for synonymous derivational markers (e.g. *warm-th* vs. *rough-ness*), authors typically talk about “suffix rivalry” (e.g. Arndt-Lappe 2014). It seems that the stereotypical expectation is that homosemous inflectional markers may not coexist side by side but must occur in complementary distribution, but it is unclear whether there is such a general distinction between inflection and derivation (cf. the existence of “overabundance” in inflection; Thornton 2011).

23 As expected given its prevalence in the textbooks, this meaning has also found its way into Wikipedia (2020): “An **allomorph** is a variant form of a morpheme, that is, when a unit of meaning varies in sound without changing the meaning.” (https://en.wikipedia.org/wiki/Allomorph, accessed 2020-04-14).

24 From the perspective of this paper, this difference is irrelevant, because all that matters is whether two phonological shapes can be regarded as variants of the same form (just as *analyse* and *analyze* are spelling variants of the same verb). Clearly, the relationships between morph variants vary in regularity, but this plays no role for the definition of the term *morph*.

25 The terminological confusion is particularly striking in Harley and Noyer’s (2003) survey article, where they first say that they want to “distinguish allomorphy from suppletion” (p. 463), but then that “there are two different types of allomorphy: suppletive and morphophonological” (p. 474).

26 Another reason why “ allomorph“ is not a good term is that it is quite opaque. Originally, the terms *morpheme*/*morph*/*allomorph* were meant to be analogous to *phoneme*/*phone*/*allophone*, but while an allophone of a phoneme is a phone, an allomorph is almost never said to be a kind of morph.

27 Note also that the members of a set of supplemorphs may themselves have morph variants, e.g. in the English morph set *-ed*/*-en* (two different homosemous past participial morphs), the first morph has the variants [d], [t] and [ɪd]. Such situations cannot be described well by an “allomorph” concept.

28 Harley (2006: 85) characterizes listemes as “minimal meaningful units of sound”, but later she posits a zero listeme (for the plural of *sheep*; 2006: 148), so that her use of listeme seems to be no different from current uses of the terms *exponent* and *vocabulary item*.

## References

[CR1] Aikhenvald A., Shopen T. (2007). Typological distinctions in word-formation. Language typology and syntactic description.

[CR2] Anderson S. R. (1992). A-Morphous morphology.

[CR3] Anderson S. R., Baerman M. (2015). The morpheme: its nature and use. The Oxford handbook of inflection.

[CR4] Arndt-Lappe S. (2014). Analogy in suffix rivalry: the case of English -ity and -ness. English Language and Linguistics.

[CR5] Bauer L., Booij G., van Marle J. (1988). A descriptive gap in morphology. Yearbook of morphology 1988.

[CR6] Bauer L. (2019). Rethinking morphology.

[CR7] Bierwisch M. (1967). Syntactic features in morphology: general problems of so-called pronominal inflection in German. To honour Roman Jakobson.

[CR8] Blevins J. P. (2016). Word and paradigm morphology.

[CR9] Bloomfield L. (1933). Language.

[CR10] Bochner H. (1993). Simplicity in generative morphology.

[CR11] Bonet E., Harbour D., Trommer J. (2012). Contextual allomorphy. The morphology and phonology of exponence.

[CR12] Booij G. E. (2005). The grammar of words: an introduction to linguistic morphology.

[CR13] Bruening B. (2018). The lexicalist hypothesis: both wrong and superfluous. Language.

[CR14] Calabrese A. (2011). Investigations on markedness, syncretism and zero exponence in morphology. Morphology.

[CR15] Carstairs A. (1987). Allomorphy in inflexion.

[CR16] Carstairs A., Booij G., van Marle J. (1988). Some implications of phonologically conditioned suppletion. Yearbook of morphology 1988.

[CR17] Carstairs-McCarthy A., Štekauer P., Lieber R. (2005). Basic terminology. Handbook of word-formation.

[CR18] Chomsky N. A. (1965). Aspects of the theory of syntax.

[CR19] Crysmann B., Bonami O. (2016). Variable morphotactics in information-based morphology. Journal of Linguistics.

[CR20] Di Sciullo A.-M., Williams E. (1987). On the definition of word.

[CR21] Dressler W. U. (1985). Morphonology.

[CR22] Embick D. (2015). The morpheme: a theoretical introduction.

[CR23] Ford A., Singh R., Martohardjono G. (1997). Pace Pānini: towards a word-based theory of morphology.

[CR24] Halle M., Marantz A., Hale K. L., Keyser S. J. (1993). Distributed morphology and the pieces of inflection. The view from Building 20.

[CR25] Harley H. (2006). English words: a linguistic introduction.

[CR26] Harley H., Noyer R. (2003). Distributed morphology. The second Glot International state-of-the-article book.

[CR27] Harris Z. S. (1942). Morpheme alternants in linguistic analysis. Language.

[CR28] Harris A. C. (2017). Multiple exponence.

[CR29] Haspelmath M. (2011). The indeterminacy of word segmentation and the nature of morphology and syntax. Folia Linguistica.

[CR30] Haspelmath M., Graf T., Paperno D., Szabolcsi A., Tellings J. (2012). How to compare major word-classes across the world’s languages. Theories of everything: in honor of Edward Keenan.

[CR31] Haspelmath, M. (2020). Towards standardization of morphosyntactic terminology for general linguistics. In: G. Arcodia et al. (Eds.), in press

[CR32] Haspelmath, M. (2021). Bound forms, welded forms, and affixes: Basic concepts for morphological comparison. In: K. Semionova et al. (Eds.), Moscow, in press

[CR33] Hockett C. F. (1947). Problems of morphemic analysis. Language.

[CR34] Jackendoff R. S. (1997). The architecture of the language faculty.

[CR35] Jackendoff R., Audring J. (2020). The texture of the lexicon: relational morphology and the parallel architecture.

[CR36] Jakobi A., Crass J., Abdoulaye B. S. (2004). Grammaire du beria (langue saharienne): avec un glossaire français-beria.

[CR37] Kiparsky P., Singh R., Desrochers R. (1996). Allomorphy or morphophonology?. Trubetzkoy’s orphan.

[CR38] Kroeger P. (2005). Analyzing grammar: an introduction.

[CR39] Lieber R. (2009). Introducing morphology.

[CR40] Lieber R., Štekauer P. (2009). Introduction: status and definition of compounding. The Oxford handbook of compounding.

[CR41] Lyons J. (1968). Introduction to theoretical linguistics.

[CR42] Matthews P. H. (1972). Inflectional morphology.

[CR43] Matthews P. H. (1974). Morphology.

[CR44] Matthews P. H. (1991). Morphology.

[CR45] Mel’čuk I. A. (1982). Towards a language of linguistics: a system of formal notions for theoretical morphology.

[CR46] Mel’čuk I. A. (2006). Aspects of the theory of morphology.

[CR47] Mugdan J. (1986). Was ist eigentlich ein Morphem?. Zeitschrift für Phonetik, Sprachwissenschaft und Kommunikationsforschung.

[CR48] Mugdan J., Müller P. O., Ohnheiser I., Olsen S., Rainer F. (2015). Units of word-formation. Word-formation: an international handbook of the languages of Europe.

[CR49] Paster M. (2009). Explaining phonological conditions on affixation: evidence from suppletive allomorphy and affix ordering. Word Structure.

[CR50] Plank F., Asher R. E. (1994). Inflection and derivation. Encyclopedia of language and linguistics.

[CR51] Plank F. (1999). Split morphology: how agglutination and flexion mix. Linguistic Typology.

[CR52] Plank F., Vincent N. (2019). Suppletion: questions for history and theory. Transactions of the Philological Society.

[CR53] Shimelman A. (2017). A grammar of Yauyos Quechua.

[CR54] Spencer A. (2013). Lexical relatedness.

[CR55] Spencer A., Luís A. R. (2012). Clitics.

[CR56] Stump G. T. (2001). Inflectional morphology: a theory of paradigm structure.

[CR57] Stump G. (2017). Rule conflation in an inferential-realizational theory of morphotactics. Acta Linguist. Acad..

[CR58] Thornton A. M., Maiden M., Smith J. C., Goldbach M., Hinzelin M.-O. (2011). Overabundance (multiple forms realizing the same cell): a non-canonical phenomenon in Italian verb morphology. Morphological autonomy: perspectives from Romance inflectional morphology.

[CR59] Trask R. L. (1999). Key concepts in language and linguistics.

[CR60] Trommer J. (2012). The morphology and phonology of exponence.

